# Effects of Modified Invasion Games on Motor Competence and Self-Assessed Physical Condition in Elementary School Students in the Physical Education Classroom

**DOI:** 10.3390/children11030337

**Published:** 2024-03-12

**Authors:** Diego Neira-Navarrete, Jacqueline Páez-Herrera, Tomás Reyes-Amigo, Rodrigo Yáñez-Sepúlveda, Guillermo Cortés-Roco, Cristian Oñate-Navarrete, Jorge Olivares-Arancibia, Juan Hurtado-Almonacid

**Affiliations:** 1 Physical Education Department, Deutsche Schule Valparaíso (DSV), Viña del Mar 2571181, Chile; d.neira@dsvalpo.cl; 2 eFidac Research Group, Physical Education School, Pontificia Universidad Católica de Valparaíso, Valparaíso 2530388, Chile; jacqueline.paez@pucv.cl (J.P.-H.); juan.hurtado@pucv.cl (J.H.-A.); 3 Physical Activity Sciences Observatory (OCAF), Department of Physical Activity Sciences, Universidad de Playa Ancha, Valparaíso 2360072, Chile; tomas.reyes@upla.cl; 4 Faculty of Education and Social Sciences, Universidad Andres Bello, Viña del Mar 2520000, Chile; rodrigo.yanez.s@unab.cl; 5 School of Education, Sport Coach, Universidad Viña del Mar, Viña del Mar 2572007, Chile; guillermo.cortes@uvm.cl; 6 Department of Therapeutic Processes, Faculty of Health Sciences, Universidad Católica de Temuco, Temuco 4813302, Chile; cristian.onate@uct.cl; 7 AfySE Group, Research in Physical Activity and School Health, School of Physical Education, Faculty of Education, Universidad de las Américas, Santiago 7500975, Chile

**Keywords:** invasion games, modified games, motor competence, physical activity, Teaching Games for Understanding

## Abstract

Modified invasion games promote the development of real and perceived motor competence. Children with higher motor competence are more likely to participate in physical activity practice and to remain in it, both in adolescence and adulthood. (1) Background: The purpose of this study is to determine the effect of modified invasion games on the real motor competence and self-assessment of the physical condition fifth-grade students from a private school in Viña del Mar, Chile. (2) Methods: 40 girls and boys with an average age of 11.47 years (SD = 0.554) participated in this study during a 12-week intervention. The MOBAK 5-6 battery was used to assess actual motor competence, the SEMOK questionnaire was used to determine perceived motor competence, the International Fitness Scale (IFIS) self-assessment questionnaire was used to assess perceived physical fitness, and the weight/size ratio was used to determine BMI. A Friedman’s nonparametric ANOVA analysis was applied to determine the effect of the intervention, in addition to an analysis of covariance (ANCOVA) to identify the influence of the covariates on motor competence. (3) Results: No statistically significant differences were established between weight, BMI, and waist circumference. There was a statistically significant difference after the intervention in the actual motor competence of object control (*p* = 0.005) and perceived motor competence of object control (*p* ≤ 0.001) (4) Conclusions: An intervention based on modified invasion games is effective for the improvement of actual and perceived motor competence of object control. It was not possible to identify a positive effect on the self-assessment of muscle strength after the intervention.

## 1. Introduction

The 17 Sustainable Development Goals (SDGs), and their 169 targets distributed in social, economic, and environmental areas, were established with the purpose of making society increasingly aware of the consequences brought about by the existing inequality in the world [1]. The pandemic caused by the coronavirus (COVID-19) in 2020, and declared in March of the same year by the World Health Organization (WHO), has exacerbated those differences, negatively impacting society in its different social systems [1]. However, the pandemic significantly affected the child population by drastically interrupting their daily activities, as well as affecting their educational processes and opportunities for socialization with their peers.

In the context of Chilean school education, it is estimated that the adjusted schooling suffered a setback of 1.3 years, losing an average of 88% of one year’s learning according to the World Bank and MINEDUC source; this was a product of the closure of schools and the restrictions implemented in the country [2,3]. Undoubtedly, this decision had consequences for the execution and development of physical education (hereinafter PE) classes. It further affected physical inactivity and increased, for example, screen time [4]. Participation in total daily physical activity (hereinafter PA) decreased by 20%, independent of pre-pandemic baseline levels [5].

In Chile, the National Survey of Physical Activity and Sports Habits 2021 (ENS) of the Ministry of Sports (MINDEP), indicated that only 5.4% of the child population between 11 and 17 years of age report PA every day of the week, while 78.5% are inactive, i.e., they have less than 3 days of PA during the week. Even more worrying is the figure for the physical activity index by region, where the Valparaíso region has a physical inactivity of 93.9%. Evidence from the report cards for Chilean schoolchildren indicates that on issues such as overweight and obesity, boys and girls obtain a score of 2.5, the same score for overall physical activity [6]. In addition to the above, the closure of schools caused students to have limited access to PA during school hours during PE classes, recess and commuting to school [7].

When the post-pandemic context is considered, [8] pointed out that there is a high risk of children being affected by prolonged states of physical inactivity, physical and mental health disorders, as well as motor aspects. In this regard, motor competence is considered as a relevant factor in the initiation or abandonment of physical activity [9] and could be a determining factor in the cause of high or low levels of overweight and obesity, which are counterproductive for the health of children; thus, children who have low levels of motor competence are those who have a higher BMI and vice versa [10]. Consequently, the higher the levels of real motor competence and self-perception, the higher the participation in physical activities [11]. In addition, the favorable development of motor competence, defined as the interaction between the neuromuscular system and the environment that facilitates the acquisition and improvement of human movement to perform activities of daily living [9], during childhood facilitates the participation of boys and girls in physical activities or sports both in and out of school [12], allowing students to become actively involved in society through games and sport [13].

Thus, educational establishments are important environments for the promotion of PA, fostering socialization and psychological attitudes (through cognitive, affective, motor and physical stimuli) in children and adolescents, and during PE classes they usually interact physically, socially, and emotionally with their peers [7]. It also provides opportunities for motor learning, and is the only instance that ensures that all children can be physically active [6]. In this sense, evidence indicates that interventions based on the teaching of modified games for understanding in school are favorable for the improvement of motor competence [14,15,16,17]. This is because they constitute a playful alternative that is located at the point of convergence between play understood as a free, self-directed activity that generates pleasure and improves well-being, and sport as a regulated, institutionalized, and codified motor situation in a competitive mode [18].

Based on the above, the execution of modified games is beneficial, from the point of view that tasks are designed for the student to understand one or more sports. For this purpose, some elements of the game are modified to allow the acquisition and knowledge of the tactical aspects of what is to be taught [19]. Modified games primarily focus on the tactical problems of the sports that need to be taught by modifying the space, rules, and resources of the game in order to highlight the tactical demands of the game. Modified invasion games are characterized by the confrontation of two teams on a shared playing field, whose scoring system is characterized by scoring in the opponent’s goal or moving a mobile to the opponent’s goal [20].

Among their main strengths, it should be noted that these games encourage student participation, as they can choose to perform in mixed groups, thus promoting the integration of both sexes. In addition, the teacher is responsible for reducing competitiveness, so as not to cause coexistence problems, where the student can act as the protagonist of the sessions by modifying and creating new games [20]. Similarly, these games favor self-perceived motor competence, as well as positive attitudes and increasing the level of PA in the classroom [21,22,23,24,25,26,27,28].

In this context, it is essential to provide procedures and information on PE programs that promote the development of motor competence, favor the development of motor skills and improve the participation of boys and girls in PA, as well as their adherence to PA both in adolescence and adulthood [29]. Likewise, guaranteeing a healthy life and promoting well-being from an early age will allow progress in achieving Goal 3 “Health and Well-being” of the 2030 Agenda for Sustainable Development [30,31,32].

Participating in modified invasion games allows children to perceive themselves as more active, their perceived motor competence positively increases, they are more motivated to engage in physical activity as well as moderate to vigorous levels of physical activity, which has positive effects [33].

Despite the above, there is a consensus within the literature that the incorporation of modified games at school has been little explored [33] in this regard. The purpose of this study was to determine the effect of modified invasion games on the real motor competence and self-assessment of the physical condition of fifth-grade students from a private school in Viña del Mar, Chile.

## 2. Materials and Methods

### 2.1. Participants

A total of 40 students (12 girls and 18 boys) with an average age of 11.47 years (SD = 0.554) participated in this study. The participants in this study belonged to one 5º primary class of a private school in Viña del Mar, Chile. The inclusion criteria for selecting participants included having a minimum class attendance of 70% and not having a pathological condition that would prevent PA. Participants with lower attendance were excluded [30]. The students participating in this study participated in compulsory physical education classes twice a week. The regular physical education classes are characterized by a traditional approach.

The application of the instruments was carried out in accordance with the ethical principles for human research as outlined in the Declaration of Helsinki (World Medical Association, 2013) and the procedural and documentation suggestions of the Research Department of Pontificia Universidad Católica de Valparaíso through its Scientific and Bioethical Ethical Committee (BIOEPUCV-H 683-2023/8 September 2023).

The authorities of the educational establishment were consulted for authorization, and informed consent, explaining the objectives and scope of the study, was obtained from the parents and/or guardians before their daughter’s participation. Throughout the development of the research, as well as in the course of writing this manuscript, practices associated with research misconduct were avoided [30,32].

### 2.2. Design and Intervention

This study is pre-experimental of a single group study with a quantitative approach [33,34,35]. The intervention was carried out for 12 weeks, with one session per week, in addition to the regular PE class, which ensured the continuation of the development of the curriculum planning established by the school. Each session lasted 60 min and was distributed in three moments. Moment 1 (beginning) lasted 15 min and was characterized by large cooperative games whose purpose was the motor activation of the participants. A second moment (development of the class) lasted 35 min, whose objective was the presentation and implementation of modified invasion games. The session ended with a closing moment or return to rest, with a duration of 10 min, in which children returned to rest and reflected on the development of the session, answering questions that encouraged discussion on values and performance during the development of the games. The intervention was guided by Learning Objective 1 of the national curriculum: “To execute collective games and sports that require making decisions and evaluating the strategies used to improve their game; for example: to apply the orientations given by the coach during the requested or regulated partial time during the game [35] for 5th grade students in the subject of PE”.

The development of the class was characterized by the implementation of modified invasion games, considering reduced game spaces, a progressive increase in the composition of the teams. Invasion games are those in which the opponents invade the rival team’s territory, trying to prevent the opposing team’s from gaining points on the scoreboard. Likewise, this type of game takes into consideration that learning tasks must be adjusted to the situational conditions demanded by sports games of this nature [36]. The proposed modified games followed the guidelines established by Valentine et al. [36], considering throws in different modalities and high intensity races, with or without a ball.

The materials used for the development of the session were sports initiation balls, sponge balls, cones, plate cones, fitballs, tennis balls, spades, hoops of different diameters. All the sessions were carried out in the gymnasium in the educational establishment during the course of the PE class.

The MOBAK test (5–6) was applied for fifth and sixth years of elementary school, translated to Spanish and validated by [37]. The assessments consist of basic motor skills of “Body Control” with four tasks (balancing, rolling, jumping, running) and “Object Control” with four tasks (throwing, catching, driving a ball with the hand, and driving a ball with the foot). The scoring for the motor tasks is dichotomously evaluated according to the successes achieved by the children. Thus, for the motor tasks related to body control, 0 points will be awarded when there are no successes, 1 point when there is one success, and 2 points when the child achieves two successes. For object control and throwing and catching tests, the scoring is as follows: 0 points when there are between zero and two successes; 1 point when the child achieves between three and four successes, and 2 points when he/she achieves between five and six successes. For the tests “driving with the hand”, “driving with the foot”, “balancing”, “rolling”, “jumping” and “running”, children have two attempts to perform. For the “throwing” and “catching” tests, they have six attempts [38]. The summary of the tasks that make up the test is shown in Table 1.

For the assessment of self-perceived motor competence, the SEMOK questionnaire (for its German acronym SElbstwahrnehmung MOtorischer Kompetenzen) [37,39] translated and validated in Spanish in Chile was used. The content of the questionnaire is shown in Table 2. This questionnaire consists of eight items related to the motor tasks of the MOBAK 5–6 test, which assesses actual motor competence. The questionnaire items are grouped like the test items, which are dimensions of body and object control. The questions are directly oriented to the MOBAK 5–6 test indicating the extent to which they consider themselves capable of performing these motor tasks. The response format the response format consists of a Likert-type scale from 1 to 5, where students express their degree of agreement with the statement made in each item (1 = strongly disagree, 5 = strongly agree).

For the evaluation of perceived physical condition, the International FItness Scale (IFIS) self-assessment questionnaire was applied. This scale is composed of five items with a Likert-type scale corresponding to the dimensions of physical condition: general physical condition, perception of cardiorespiratory physical condition, perception of muscular strength, speed/agility and flexibility. The response possibilities are as follows: very bad (1), bad (2), acceptable (3), good (4), and very good (5).

Body Mass Index (BMI) was measured using the following equation: (kg/m^2^) division of weight in kilograms by the square of height in meters. The SECA 206 portable measuring rod was used to obtain height, weight was obtained using a SECA Aura 807 digital scale, and waist circumference was obtained with the SECA 201 tape measure. The BMI Z-score formula recommended by the WHO was used to measure children between 5 and 19 years of age [40].

All measurements were performed in the PE class and were applied by the teacher responsible for the research, as well as by two PE teachers from the educational establishment, who were trained for the application of each test.

### 2.3. Recording of Information and Statistical Analysis

Data were entered in a Microsoft 365^®^ Excel 2019^®^ spreadsheet along with age in months, date of birth, biological sex, results of actual motor competence tests, self-perceived motor competence, IFIS questionnaire, and anthropometric measurements. The data are shown in averages and standard deviations; 95% confidence intervals were also considered. The SPSS^®^ version 24 statistical program was used for data analysis. The Shapiro–Wilk test was used to analyze the normality of the data. The repeat-measures ANOVA was then applied to identify the effects of the intervention, and the Bonferroni post hoc test was used to determine the *p*-value. Finally, the effect size of the intervention was calculated through Cohen’s d test, being classified as follows: no effect (<0.2), small (>0.2 to 0.5), medium (>0.5 to 0.8) and large (≥0.8) effects (Cohen, 1988 [41]). In all tests, a statistically significant value was considered as *p* < 0.05. In addition, an analysis of covariance (ANCOVA) was applied, adjusting the variables to size and time (PRE and POST), and no significant effects of this on the study variables were found (*p* < 0.05)

## 3. Results

Table 3 shows the characterisation data of the participants. Forty children in their fifth year of basic education participated in the present study. Regarding basic anthropometric measures, the mean age was 11.47 (SD = 0.554) during the intervention. The descriptive data (Mean and SD) and the repeat-measures ANOVA analysis used did not allow statistically significant differences to be established between weight, BMI, size, and waist circumference.

Table 4 shows that for the variables associated with the self-assessment of general fitness and physical abilities, there was an increase in the means for general fitness, cardiorespiratory fitness, speed/agility and flexibility between one intervention and the other. Only muscular strength showed a decrease in means after the intervention. However, no statistically significant differences were observed for any variables after the intervention. On the other hand, it is not possible to see any statistical effect of the intervention on each of the self-assessed fitness variables.

Table 5, which shows the effect of the intervention on the real and self-perceived motor competence of the participants, shows that in both the object control and body control tests, there was an increase in the scores after the intervention. However, only in the object control tests was there a statistically significant difference (*p* = 0.005). As for the self-perceived motor competence, there is a coincidence as to what happened with the real motor competence, that is, there was an increase in the self-perception of body control and in the tests associated with body control; however, only in the latter was there a statistically significant difference (*p* = 0.001) after the intervention. Similarly, it is possible to note that after the intervention, there was an increase in the total self-perceived motor competence of the participants, showing a statistically significant difference (*p* = 0.050).

Looking at the results after the Cohen’s d test, it can be seen that after the intervention there was a small effect on the tests of total perceived motor competence (d = 0.382) and perceived motor competence of body control (d = 0.380). Meanwhile, the intervention had a medium effect on the actual motor competence of body control (d = 0.563) and on the actual motor competence of object control (d = 0.542). The intervention had a greater effect on the perceived motor competence of object control (d = 0.912).

## 4. Discussion

The purpose of this study was to determine the effect of modified invasion games on the actual motor competence and self-assessment of the physical fitness of fifth-grade elementary school students. The results of this study indicate that after the intervention, there was an increase in the self-assessment of general physical fitness, as well as cardio respiratory fitness, speed/agility, and flexibility. However, in the physical capacity of strength, there was a decrease in the self-assessment of strength after the intervention of modified invasive games. Our results are related to those reported in an intervention proposal based on the Teaching Games for Understanding (hereinafter TGFU) model, mainly used in invasive sports games, reporting that participants significantly increased the level of light, moderate and vigorous PA, as well as the average step count during the PE class [42,43]. Positive values are also reported after the intervention, in tests of total distance traveled, maximum speed threshold, and amount of sprinting during the course of the games [44]. A similar situation occurred in an intervention, in which the experimental group, after an intervention of modified games with reduced groups, increased scores in tests of cardiorespiratory fitness, heart rate, flexibility and speed [33].

However, for the above, there is also evidence [45] that an intervention based on TFGU, reports a lower physiological response than an intervention of a contextualized sports literacy model, as the modified games mainly focus on tactical/technical reflection during the development of the game.

It would seem that the increases in the self-assessment of general physical fitness, cardiorespiratory fitness, and PA levels are explained by the fact that the invasion games in the TFGU teaching model grant greater freedom of movement and report greater commitment in the development of the games [43], ultimately allowing a reduction in the inactivity of boys and girls in the PE class [46].

Regarding real and perceived motor competence, our results indicate that the intervention based on modified invasion games reports positive results in the tests of object control, body control, as well as in the perception that the participants had in both dimensions of motor competence. A quasi-experimental design intervention with 41 students based on the TGFU teaching model coincides with our results, indicating that after 16 weeks, participants reported improvements in perceived motor competence, independent of the dimension [16]. In turn, there is evidence that a TGFU model intervention is more effective in improving perceived motor competence than variables such as autonomous motivation and intention to be physically active [47]. Evidence also indicates that when comparing a group of students whose intervention was based on instructional techniques with a group that participated in comprehension game sessions, the latter reported higher scores at the end of the intervention in object control tests, mainly in those involving passing a mobile or receiving it [48]. This situation coincides with the results of our study, where the participants, at the end of the intervention, report a statistically significant difference, both in the actual motor competence of object control and in the perceived competence of the same dimension, compared to the actual and perceived motor competence of body control.

Likewise, our results indicate that after the intervention of modified games, only the real motor competence of object control and the perceived motor competence of the same dimension increased in scores with a statistically significant difference between pre- and post-test. This finding seems to be interesting, since there is evidence that greater real and perceived motor competence in object control favors greater adherence to the practice of PA in adolescence, given that the type of events in which young people are involved is diversified [49,50]. Similarly, if we consider the results of this study regarding the improvement in the self-assessment of the participants’ general physical condition and cardiorespiratory capacity, after the intervention, it is possible to note that the model of games for understanding is also beneficial for the health of the participants [49]. In this context, given the particularities of the modified invasion games in the TGFU teaching model, it is possible to expect improvements in motor competence, both real and perceived; mainly because of the intensity that the modified games possess. Thus, it becomes an excellent opportunity to improve health-related issues [51,52].

Among the limitations of the study, it is possible to note the lack of devices that allow direct assessment of the level of PA. Likewise, we consider that it is important to improve the study by including a control group and an experimental group, which would allow us to determine with greater precision the efficacy of the intervention. Finally, another limitation of the study is the implementation of the intervention for one session per week, which is mainly due to administrative aspects of the educational establishment.

## 5. Conclusions

It is concluded that the effect of the implementation of 12 classes of modified invasion games designed under the Teaching Games for Understanding model has a positive effect on real and perceived motor competence. Likewise, an intervention of this nature provokes a greater effect on the real and perceived motor competence of object control, finding statistically significant differences in comparison to the body control dimension of both real and perceived motor competence. Similarly, after the intervention, favorable results were reported in the self-assessment of general physical condition, cardiorespiratory capacity, speed/agility and flexibility. However, after the results, it is evident that the intervention must comply with other conditions and characteristics in order to have a positive impact on the self-evaluation of strength.

It is suggested to continue investigating the effect of incorporating modified invasion games under the Teaching Games for Understanding model in the PE class, due to the impact they could have on increasing the levels of PA, as well as on the adherence and permanence in practices of this nature, both in adolescence and adulthood. Promoting PA in schools, together with teaching strategies that encourage an active and healthy life, contribute more effectively to the development of a healthy society, with quality of life and wellbeing.

Among the strengths of the study, we identified the proposal of modified invasion games as a pedagogical resource to favor the motor competence of boys and girls, as well as a means for the development of healthy competences at school.

As for future lines of research that have been generated from the study, it is essential to continue gathering information on the effect of modified invasion games on healthy lifestyles and the level of PA, since the literature is emphatic in pointing out that there is significant evidence for the benefits it has in tactical thinking, as well as prosocial and attitudinal development, in the PE class. Likewise, we must continue investigating how motor competence can be a predictor of adherence PA practice in adulthood.

## Figures and Tables

**Table 1:** Descriptive summary of the dimensions and items of the MOBAK 5–6 battery.

Dimensions	Items	Description
Object Control	Launch	Throw, from 3.0 mts. away from the wall, a ball to try to hit a circle marked on the wall.
	Catch	Throwing a ball into the air and catching it before it falls to the ground.
	Driving with the hand	Hand-drive a No. 3 basketball through a lane (7.5 mts. × 1.4 mts.) with four obstacles.
	Driving with the foot	To drive with the foot a futsal ball N° 4 through a lane (7.5 mts. × 1.4 mts.) with four obstacles.
Body Control	Balance	Walk backwards and forwards over an inverted swinging bench with two 6.0 cm high obstacles.
	Roll	Perform a forward somersault on a mat placed on two gymnastic crates.
	Jump	Jumping rope in place, continuously, for 20 s.
	Run	Run frontally and diagonally in a rectangle (2.0 m × 4.0 m).

Source: own elaboration.

**Table 2:** Descriptive summary of the items in the SEMOK questionnaire.

Dimensions	Motor Task	Affirmation
Object Control	Launch	I can throw a ball to accurately hit a target on the wall.
	Catch	I can catch a tennis ball safely
	Driving with the hand	I can drive a basketball
	Driving with the foot	I can drive a soccer ball
Body Control	Balance	I can walk (forward and backward) on a balance beam that swings.
	Roll	I can do a forward somersault with a pre-jump.
	Jump	I can jump a rope changing pace
	Run	I can run by changing direction

Source: own elaboration.

**Table 3:** Characteristics of the participants.

Variables	Average y SD	95% Confidence Interval		Average y SD	95% Confidence Interval		ES	*p*
		Lower	Upper		Inferior	Superior		
	PRE (*n* = 40)			POST (*n* = 40)				
Age	11.47 ± 0.554	11.30	11.65	11.47 ± 0.554	11.30	11.65	0.000	1.000
Weight	46.60 ± 9.789	43.47	49.73	47.89 ± 10.492	44.53	51.25	0.127	0.571
Size	153.41 ± 7.309	151.08	155.75	155.74 ± 8.152	153.14	158.35	0.301	0.183
Wcir.	66.65 ± 8.356	63.98	69.32	68.10 ± 9.586	65.03	71.17	0.163	0.473
BMI	19.72 ± 3.516	18.60	20.84	19.67 ± 3.633	18.50	20.83	0.015	0.946

BMI: body mass index; Wcir.: waist circumference; SD: standard deviation; *p* value < 0.05.

**Table 4:** Physical capacity variables, measured by the IFIS self-assessment questionnaire before and after the intervention of modified invasive games.

Variables	Average y SD	95% Confidence Interval		Average y SD	95% Confidence Interval		ES	*p*
		Lower	Upper		Inferior	Superior		
	PRE (*n* = 40)			POST (*n* = 40)				
Physical condition	3.90 ± 1.033	3.57	4.23	4.03 ± 0.891	3.74	4.31	0.129	0.566
Cardio-respiratory CF	3.73 ± 1.086	3.38	4.07	3.83 ± 0.874	3.55	4.10	0.101	0.653
Muscula strength	3.83 ± 0.931	3.53	4.12	3.70 ± 1.043	3.37	4.03	0.126	0.575
Velocity/agility	4.17 ± 0.958	3.87	4.48	4.22 ± 0.832	3.96	4.49	0.056	0.803
Flexibility	3.38 ± 1.275	2.97	3.78	3.42 ± 1.338	3.00	3.85	0.068	0.865

SD: standard deviation; *p* value < 0.05.

**Table 5:** Variables of real and self-perceived motor competence, before and after the intervention of modified invasion games.

Variables	Average y SD	95% Confidence Interval		Average y SD	95% Confidence Interval		ES	*p*
		Lower	Upper		Inferior	Superior		
	PRE (*n* = 40)			POST (*n* = 40)				
Full control of MOBAK objects	5.30 ± 1.814	4.72	5.88	6.28 ± 1.840	5.69	6.86	0.542	0.018
MOBAK total body control	6.03 ± 1.641	5.50	6.55	6.85 ± 1.252	6.45	7.25	0.563	0.014
SEMOK body control	3.91 ± 0.755	3.66	4.15	4.21 ± 0.848	3.94	4.48	0.380	0.093
SEMOK object control	3.79 ± 1.055	3.45	4.13	8.17 ± 1.124	7.82	8.53	0.912	<0.001
SEMOK total	3.85 ± 0.770	3.60	4.09	4.15 ± 0.805	3.89	4.40	0.382	0.092

SD: standard deviation; *p* value < 0.05.

## Data Availability

The data presented in this study are available on request from the corresponding author. The data are not publicly available due to privacy or ethical restrictions.
